# Effect of altered haemodynamics on the developing mitral valve in chick embryonic heart

**DOI:** 10.1016/j.yjmcc.2017.05.012

**Published:** 2017-07

**Authors:** Kar Lai Pang, Matthew Parnall, Siobhan Loughna

**Affiliations:** School of Life Sciences, Medical School, University of Nottingham, Nottingham NG7 2UH, UK

**Keywords:** Haemodynamics, Cardiac valve development, Mitral valve, Valve defect, Valvulogenesis, Outflow tract banding

## Abstract

Intracardiac haemodynamics is crucial for normal cardiogenesis, with recent evidence showing valvulogenesis is haemodynamically dependent and inextricably linked with shear stress. Although valve anomalies have been associated with genetic mutations, often the cause is unknown. However, altered haemodynamics have been suggested as a pathogenic contributor to bicuspid aortic valve disease. Conversely, how abnormal haemodynamics impacts mitral valve development is still poorly understood. In order to analyse altered blood flow, the outflow tract of the chick heart was constricted using a ligature to increase cardiac pressure overload. Outflow tract-banding was performed at HH21, with harvesting at crucial valve development stages (HH26, HH29 and HH35). Although normal valve morphology was found in HH26 outflow tract banded hearts, smaller and dysmorphic mitral valve primordia were seen upon altered haemodynamics in histological and stereological analysis at HH29 and HH35. A decrease in apoptosis, and aberrant expression of a shear stress responsive gene and extracellular matrix markers in the endocardial cushions were seen in the chick HH29 outflow tract banded hearts. In addition, dysregulation of extracellular matrix (ECM) proteins fibrillin-2, type III collagen and tenascin were further demonstrated in more mature primordial mitral valve leaflets at HH35, with a concomitant decrease of ECM cross-linking enzyme, transglutaminase-2. These data provide compelling evidence that normal haemodynamics are a prerequisite for normal mitral valve morphogenesis, and abnormal blood flow could be a contributing factor in mitral valve defects, with differentiation as a possible underlying mechanism.

## Highlights

• Altered haemodynamics leads to defective mitral valve primordia in post-EMT heart.
• Cushion mesenchyme markers were aberrantly expressed in abnormal valve primordia.
• Decreased expression of ECM proteins were seen in defective mitral valve primordia.
• Shear stress gene differentially expressed in defective mitral valve primordia.
• Differentiation is the likely mechanism causing the dysmorphic valve phenotype.

## 1 Introduction

A mature heart has four cardiac valves; pulmonary and aortic valves (semilunar valves) and tricuspid (TV) and mitral valves (MV) (right and left atrioventricular valves, respectively). These valves ensure unidirectional blood flow to both pulmonary and systemic systems. Aberrant developmental mechanisms can occur and give rise to congenital valve defects [1]. Congenital valve malformations have a prevalence of 2% of live births and occur in a number of genetic syndromes such as Marfan syndrome and trisomy 21, and can happen in isolation or in conjunction with other heart defects including hypoplastic left heart syndrome and Tetralogy of Fallot [1], [2], [3]. Congenital MV anomalies were detected in 0.5% of 13,400 subjects in an echocardiographic study and are susceptible to subtle developmental abnormalities [4]. Although MV anomalies such as myxomatous valve disease, MV atresia, stenosis and prolapse often have an unknown aetiology, they have also been associated with mutations in fibrillin-1, filamin A and *NOTCH1* [5], [6], [7], [8], [9]. It is known that haemodynamics plays an important role in normal valve morphogenesis and is associated with shear stress [10], [11], [12], [13]. In recent years, the alteration of haemodynamics has been proposed to be a pathogenic contributor to valve defects [14], [15]. Absent atrioventricular (AV) valves were seen upon occlusion of haemodynamics in zebrafish [10], whereas constriction of blood flow by placing a ligature around the outflow tract (OFT-banding) increased mitral-aortic valve separation and valve regurgitation as well as affected OFT valve development in chick [16], [17], [18], [19]. With regards the tricuspid valve, immature or abnormal TV valves were found upon morphological analysis of banded hearts in the chick [20], [21].

The heart is the first organ to form as a linear tube and it consists of outer myocardium and inner endocardium which sandwich a layer of extracellular matrix (ECM) called cardiac jelly [3]. During looping of the chick heart tube at HH14, valvulogenesis initiates with ECM swellings that expand at the atrioventricular canal (AVC) and OFT, forming the endocardial cushions (EC), which act as primitive valves [22], [23]. ECs form when a subset of endocardial cells lose intercellular adhesion and migrate into AV cushion mesenchyme, by endocardial-mesenchymal transformation (EMT) [22]. Subsequent growth and septation take place as the superior and inferior EC fuse to produce the central mesenchymal mass [24], [25]. This mass fuses with the dorsal mesenchymal protrusion and mesenchymal cap at the inferior part of the atrial septum primum at HH24 and the upper portion of the ventricular septum at HH29/30 to fully septate the primitive atria and ventricles into four chambers [25], [26], [27], [28], [29].

During post-EMT (from HH26), distal outgrowth and maturation of the ECs take place via migration, apoptosis and proliferation with the expression of ECM proteins [30]. In addition, valve-associated fibrous structures such as chordae tendinae form and the cushion mesenchymal cells differentiate into valvular interstitial fibroblasts [31]. The remodelled AV valve primordia eventually develop into mature structures with three stratified layers containing different ECM components; atrialis (fibrillin-1, fibrillin-2, fibronectin), spongiosa (perlecan, aggrecan) and fibrosa (collagen type I and III, tenascin) [32], [33]. Each layer has a specified role in withstanding the mechanical load in the heart [32], [33], [34], [35]. With regards the valve leaflets, the septal leaflets of the TV form by delamination from the muscular ventricular septum, whereas the MV septal leaflets form by elongation and protrusion of the cushion mesenchyme into the ventricular lumen [36], [37]. The lateral leaflets form due to proliferation of the AV mesenchyme from the lateral myocardial wall [38].

Herein, our aim was to characterize the effect of altered haemodynamics on the developing MV at the morphological and molecular level. In the present study, we describe dysmorphic mitral valve primordia upon the alteration of haemodynamics using OFT-banding in the chick. In addition, the deformed HH29 primordial valves aberrantly expressed shear stress responsive genes alongside genes encoding ECM proteins, and a decrease in apoptosis was seen, factors possibly linked with failure of differentiation. Dysregulation of ECM proteins was further demonstrated in primordial valve leaflets at later stage of valve development, with a concomitant decrease of ECM cross-linking enzyme, transglutaminase-2. We provide evidence of novel roles for haemodynamics on the developing MV during post-EMT, and suggest abnormal blood flow as a potential pathogenic contributor to valve defects.

## 2 Materials and methods

### 2.1 Outflow tract banding

White fertile chicken eggs (*Gallus gallus*; Dekalb white strain; Henry Stewart, UK) were incubated at 38 °C in a humidified atmosphere under constant rotation for 4 days until Hamburger and Hamilton stage 21 (HH21) [39]. After windowing, the inner shell membrane was removed to expose the heart. OFT-banding was performed as described using 10-0 nylon suture [19], [21], using a double overhand knot snug around the OFT. Sham-operated embryos underwent the same procedure but had the suture removed immediately. Unoperated embryos were windowed, staged, and reincubated. All eggs were sealed and incubated for an additional 2–5 days until HH26-35. Animal work was performed in accordance with national (UK home office) and institutional regulations and ethical guidelines.

### 2.2 Embryo isolation

OFT-banded and control (sham and unoperated) embryos were isolated at HH26, HH29 and HH35 and external analysis was performed. Hearts were then fixed in 4% PFA, washed twice in PBS, dehydrated in an ethanol series and wax-embedded in a transverse orientation. Unless otherwise specified, serial 8 μm sections were taken (DSC1 microtome, Leica, Germany), dewaxed and rehydrated. For histological studies, sections were stained with Alcian Blue (Sigma, UK) for 15 min at room temperature (RT) followed by Mayers haemalum (Raymond Lamb, UK). Images were acquired by a slide scanner (Nanozoomer 2.0-HT, Hamamatsu, Japan).

### 2.3 Stereology and morphometric measurement

Systematic random sampling [40] was used to assess tissue proportions throughout HH29 hearts (control [unoperated and sham] and OFT-banded; n = 12 controls and n = 7 OFT-banded). A 96-point grid was placed over every fifth section on the AV valve region which comprises the primordial AV septal and lateral leaflets in both right and left sides of the heart. Each point on the AV valve tissue was counted (223, 218 and 272 points for sham, unoperated and banded, respectively). Average tissue proportions were calculated and tested for statistical significance (see below). For HH29 valve region morphometric measurement, the endocardial cushion (μm^2^) and the raw length primordial MV septal leaflet (μm) were measured on every third section using nanozoomer viewing software (NDP.view2). Relative cushion area was determined by dividing the cushion area of OFT-banded hearts by that of control heart; valve length was determined using the same procedure [41]. For quantification of embryo size, crown lump length, eye and eye lens diameter measurements were taken from HH29 embryos (n = 20) as previously described [42].

### 2.4 In situ hybridization

Riboprobes for shear stress responsive genes (Krüppel lung factor 2, *KLF2*; endothelin 1, *EDN1*) and ECM markers of endocardial cushion (versican, *VCAN*; T-box 20, *TBX20*) were designed in house. Aggrecan, periostin and positive control *GAPDH* sequences were used as previously reported [43], [44], [45]. Primers are listed in Table 1. All riboprobes were made with incorporation of Digoxigenin (DIG)-UTP (Roche) following manufacturer's instructions. *KLF2*, *EDN1*, *TBX20* and aggrecan antisense probes were synthesized with T7 polymerase from plasmids linearized with *Spe*I. *VCAN* and *GAPDH* antisense probes were synthesized with SP6 polymerase from the plasmid linearized with Ncol, with periostin synthesized with SP6 polymerase from a plasmid linearized with *Pvu*II.

**Table 1:** Primers pairs and annealing temperatures with amplicon size designed for riboprobe synthesis.

Primer	GenBank accession number	Primer sequence (5′ to 3′)	Product size (bp)	Annealing temp (°C)
		Forward/reverse		
*KLF2*	NM_001318423	ATGGTGAATGACTGCCACAC	515	62
		ATGTGCCGCTTCATGTGC		
*EDN1*	XM_418943.4	GAAGTGAACGCCGCATCG	528	62
		GCTTTTCCAGATGCTTTGCC		
*TBX20*	NM_204144.1	AGATATGCCTACCACCGCTC	783	62
		ATGGTACCTTGGCATGTGGA		
Aggrecan	NM_204955.2	CTGCGTTCCCTGAGATTAC	963	62
		TTGCCAGGTCGATCTCAC		
Periostin	NM_001030541	TAATGCTCTCCACCACCACA	1001	57.8
		TCTGCTGGCTTGATGATTTG		
*VCAN*	NM_204787	GCAACAAACAATACAGCCCC	508	62
		CTCTCTCAGCCGTATCCCAG		
*GAPDH*	NM_204305.1	GGGCTCATCTGAAGGGTGGTGCTA	810	62
		GTGGGGGAGACAGAAGGGAACAGA		

Paraffin-embedded neighbouring sections (8 μm) from HH26 and HH29 hearts were mounted on Superfrost Plus microscope slides (Fisher Scientific) and prepared for in situ hybridization as previously described [46] with some modifications. After deparaffinization, sections were treated with 20 μg/mL proteinase K for 8 min at RT. After terminating proteinase K reaction with glycine (2 mg/mL) and post-fixation with 4% PFA, sections were acetylated 10 min in 0.25% acetic anhydride in 0.1 M triethanolamine pH 8.0. Sections were pre-incubated with the hybridization mixture (50% formamide, 5 X SSC, 1 X Denhardt's solution, 10% Dextran sulfate, 0.1% Tween-20, 50 μg/mL Heparin, 1 mg/mL tRNA) for 2 h at 67 °C, and reacted overnight at 67 °C with DIG-label RNA probes (4–7 μL) added to the hybridization mix. After post hybridization washes and blocking steps, the sections were incubated overnight at 4 °C with alkaline phosphatase-conjugated DIG antibody (1:2000, Roche). Sections were stained with BM purple at RT for colour development and mounted for imaging using Axioplan microscope (Zeiss).

### 2.5 Quantitative PCR analysis

Snap frozen hearts and isolated AV canals were placed at − 80 °C upon harvesting at HH29 and HH35. Total RNA was extracted using Trizol reagent (Sigma), and treated with RNase-free DNase I (Qiagen), followed by first strand cDNAs synthesis. 1 μg of RNA was used in each reaction. Each cDNA sample was diluted 4 times with nuclease-free water before determination of the dynamic range of sample concentrations for the qPCR experiment. Stock was diluted 1:33 and 4uL of this was added to final relative quantitation experiment. This dilution represents values mid-range of the slope in the standard curve optimisations. Optimization of primer concentrations for relative quantitation and qPCR were performed using the Applied Biosystems 7500 Fast Real-time PCR system as previously described [47]. Efficiencies of all gene reactions were 94.2–106%, with r^2^-values > 0.98. All samples were run in triplicate within each PCR experiment. Each 20 μL PCR reaction mixture consisted of 10 μL of iTaq™ universal SYBR® Green supermix (1 ×), 0.5 or 0.75 μL of each forward and reverse primer (250 or 375 nM) for *KLF2*, periostin, aggrecan, *EDN1*, *TBX20*, transglutaminase 2 (*TGM2*), fibrillin 2 (*FBN2*), collagen type III alpha 1 chain (*COL3A1*), tenascin C (*TNC*) and 4 μL of diluted cDNA template dependant on individual genes (Table 2). Relative gene expression was quantified against *GAPDH* and *EEF1A1* as reference genes. qPCR was performed on isolated AV canals for *KLF2* at HH29 and *TGM2*, *FBN2*, *COL3A1*, *TNC* at HH35. Otherwise, the qPCR was done in whole hearts for the remaining genes at HH35.

**Table 2:** Primers pairs used for qPCR.

Primer	GenBank accession number	Primer sequence (5′ to 3′)	Primer concentration (nM)	Product size (bp)
		Forward/reverse		
*KLF2*	NM_001318423	GCTTCTACCAGACAAACCCG	250	233
		CAGGACTGGCCCATAACTGT		
*EDN1*	XM_418943.4	GATGTGCCAGCCAGAGAGACAA	375	180
		CAGCCTCCAGCCTCTTCATTTTC		
*TBX20*	NM_204144.1	AGATATGCCTACCACCGCTCCT	375	178
		TGATGTGGCCATGCTGATCCA		
Aggrecan	NM_204955.2	CGGCATCTGGACAAGAGACAGA	250	165
		CTCCATTCAGACAGGGGCTTGA		
Periostin	NM_001030541	AGAACCTGATCTCATGGCAACCA	250	183
		GGGTTGTAAAACGTCAGTGGAATCT		
*TGM2*	NM_205448.1	CCAGCCCCACATGGAACAGA	375	128
		CCACGCTGTCACCAGTCTCA		
*GAPDH*	NM_204305.1	AGACGGTGGATGGCCCCTCT	375	263
		ACGGCAGGTCAGGTCAACAACA		
*EEF1A1*	NM_001321516.1	GCTCTAACATGCCCTGGTTCAAG	375	188
		TGGCTTCAGGACACCAGTTTC		
*FBN2*	XM_004949379.2	GCCCATGTGAGCGGTGTGAA	250	194
		CACTCGCCCTGTTCCATCCA		
*COL3A1*	NM_205380	CTGGAAGGGCAGGGAACAAC	250	249
		GGCATGGCTCTGGTTTCCAA		
*TNC*	NM_205456.4	GCTGAGGGTGGATGGCTACAG	250	168
		CCCATCAGATTGACTCGGTGACA		

### 2.6 Apoptosis

ApopTag Peroxidase In Situ Apoptosis Detection Kit S7100 (Millipore, USA) was used to indicate apoptotic cells in accordance with manufacturer's instructions on 5 μM serial sections. Imaging was performed using Zeiss Axio Scan Z1. Systematic random sampling [40] was utilised to count positive cells against total cell count in the endocardial cushions, primordial MV and TV septal and lateral leaflets to calculate proportions of apoptotic cells for statistical analysis (see below). A total of 224,749 cells were identified as either apoptosis ‘positive’ or ‘negative’ in OFT-banded hearts at HH29 (n = 5 per group).

### 2.7 Fibrosis and immunohistochemical studies

HH35 OFT-banded and control hearts were serially sectioned (8 μm), dewaxed and rehydrated in graded ethanol series and water, with neighbouring sections placed on adjacent slides to allow for the analysis of different stains. One set of the sections were mordanted in Bouin's fixative for 1 h at 56 °C and stained using Trichrome Stain (Masson) Kit (Sigma) based on manufacturer's instructions. Sections were differentiated in 1% acetic acid followed by dehydration. Images were taken using an Axioplan microscope (Zeiss).

For fluorescence immunohistochemistry, antigen retrieval was performed (microwaving for 10 min in 10 mM citric acid buffer pH 6.0) on three sets of neighbouring sections. They were blocked in 10% goat serum in 1% BSA/PBS for 2 h at RT with primary antibody incubation overnight at 4 °C. Primary antibodies used were JB3 (fibrillin-2; 1:50; DSHB, USA) [48], M1-B4 (tenascin; 1:50; DSHB) and 3B2 (type III collagen; 1:25; DSHB). Sections were incubated with secondary antibody (Alexa 488; 1:100, Molecular Probes; A21121) for 1 h at RT. Nuclei were counterstained with DAPI (1:1000; Sigma; D9542) and imaging was performed (Leica DMIRE2). Measurement of the fluorescent intensity (represented by grey values of all pixels within region of interest) was performed in the primordial MV septal leaflet using MetaExpress software (n = 5 per group). For the spatial-temporal expression profiles studies, unoperated control embryos (n = 2 per stage) at HH21, HH26, HH29 and HH35 were isolated, wax-embedded and sectioned as described above. Immunohistochemistry was performed using the antibodies described above.

### 2.8 Statistics

All data was analysed by parametric tests as the Shapiro-Wilk test values equal *P* > 0.05. Levene's test was used to assess for equality of variances. If *P* < 0.05, two-tailed assuming equal variances was used to test for differences between group means on all samples (n ≥ 3) in all experiments (SPSS V21 (SPSS, USA)); *P* < 0.05 were considered to be significant.

## 3 Results

### 3.1 Alteration of haemodynamics results in valve morphogenetic abnormalities

**Fig. 1:**
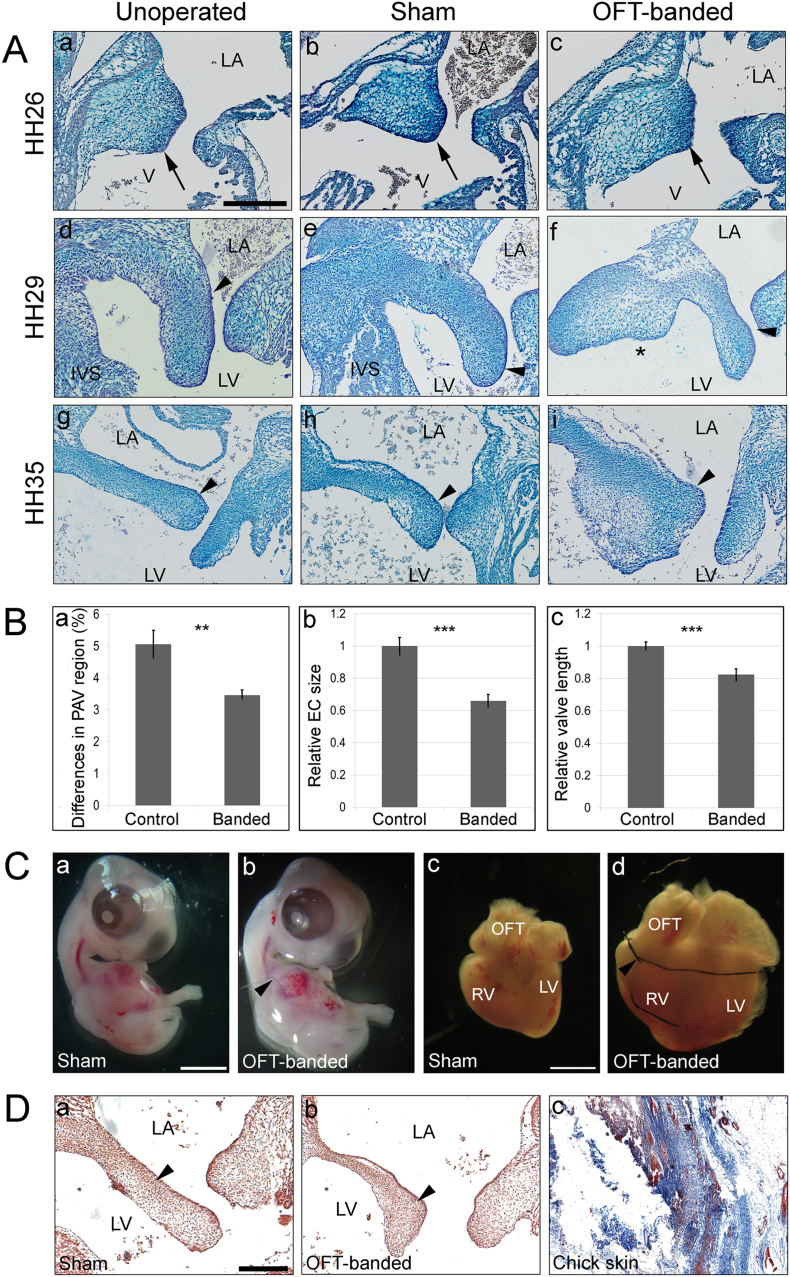
Defective mitral valve in OFT-banded hearts. A. Alcian blue stained sections from unoperated (a, d, g), sham (b, e, h) and OFT-banded (c, f, i) hearts at HH26 (n = 5 per group; a–c), HH29 (n = 31 for OFT-banded and n = 37 for controls; d–f) and HH35 (n = 7; g–i). Valve morphology at HH26 appeared comparable in all hearts (a–c; denoted by arrow). In OFT-banded hearts, defective primordial mitral valve appeared either smaller (arrowhead in f) or presented with nodular thickening (arrowhead in i) at HH29 and HH35 in comparison to controls (d, e, g, h; denoted by arrowhead). Asterisk in Af denotes the absence of the interventricular septum (IVS) in the OFT-banded hearts. B. Stereology revealed the primordial AV region was proportionally smaller in the OFT-banded hearts than controls at HH29 (a). Relative cushion area (b) and relative valve length (c) were smaller and shorter in banded compared to controls. Student's *t*-test (two-tailed). Values are mean ± s.e.m. (n = 7 for OFT-banded and n = 12 for controls) (**P < 0.01; ***P < 0.001). C. Representative images of sham (a) and OFT-banded embryos (b) at HH29, showing the size is similar between both groups. OFT-banded hearts (d) appeared to be larger than the controls (c) at HH29. Arrowhead denotes the ligature around the outflow tract region in the banded hearts. D. Histological analysis using Masson's Trichrome staining revealed no fibrosis (arrowhead indicates blue staining absent) in the primordial MV septal leaflets at HH35 between sham (a) and banded hearts (b) (n = 5). Chick skin shown as a positive control (c). LA, left atrium; LV, left ventricle; OFT, outflow tract; RV, right ventricle; V, ventricle. Scale bars: 250 μm for Aa-i, 1000 μm for Ca-d, 80 μm for Da-c. (For interpretation of the references to colour in this figure legend, the reader is referred to the web version of this article.)

To investigate the role of abnormal haemodynamics in the development of the primordial AV valve and its alignment with other septal components, OFT-banding of the chick embryonic heart was performed at HH21 (AVC EC is a localized protrusion of cardiac jelly; atrial and ventricular septation are ongoing). Harvesting was performed at different stages of post-EMT primordial valve development, at HH26, HH29 and HH35. Histological analysis revealed that the dysmorphic valve primordial leaflets were seen in 19 of the HH29 banded hearts (n = 31) and in most HH35 banded hearts (n = 6/7), compared to no abnormalities observed in controls at either HH29 (n = 37) or HH35 (n = 7) respectively. Morphologically, the valve leaflets appeared smaller (arrowhead in Fig. 1Af compared to d, e) or presented with a nodular thickening at the distal end of the valve leaflet (arrowhead in Fig. 1Ai compared to g, h) in banded hearts. Also, the hearts which had dysmorphic AV valves also displayed a ventricular septal defect at HH29 (asterisk in Fig. 1Af) and HH35 (data not shown). In contrast, the AV cushion was fully fused with the interventricular septum (IVS) in all controls (Fig. 1Ad, e for HH29) and HH35 (data not shown). The atrial septum and endocardial cushion at HH26 appeared morphologically normal in banded and control hearts (n = 5 per group; arrow in Fig. 1Aa–c).

Stereological analysis (at HH29) showed that the primordial AV valve in controls represented 5.06 ± 0.39% (n = 12), whereas the valve region in the OFT-banded hearts accounted for 3.46 ± 0.11% (n = 7), a decrease of 31.62% (two-tailed Student's *t*-test; *P* = 0.002; Fig. 1Ba). This was supported by the morphometric measurement of the cushion area and raw primordial valve length at HH29; a smaller EC area was seen, which was 66% of the size of the controls (*P* < 0.001; Fig. 1Bb). Further, shorter primordial MV leaflets (Fig. 1Af), which were 82% of the length of controls (*P* < 0.001; Fig. 1Bc), were seen. However, the overall size of the embryo remained comparable between two groups (Fig. 1Ca, b), as shown by the similar crown rump length (3.74 ± 0.25 cm and 3.80 ± 0.27 cm in banded and sham, respectively; *P* > 0.05). Further, the eye lens diameter presented 0.22 ± 0.01 cm in banded hearts and 0.23 ± 0.01 cm in sham (*P* > 0.05), and the eye diameter measured 0.95 ± 0.06 cm in banded and 0.97 ± 0.06 cm in sham (*P* > 0.05) (two-tailed Student's *t*-test). Upon Masson's Trichrome staining, deposition of collagen was not found in any of the primordial AV leaflets and EC in HH35 control or OFT-banded hearts (n = 5 per group; arrowhead in Fig. 1Da, b; chick skin positive control Fig. 1Dc).

### 3.2 Aberrant expression of *TBX20* and aggrecan in endocardial cushion and valve primordia of HH29 banded hearts

To further characterize the valve phenotype, the *TBX20* transcription factor and its downstream ECM valve markers (aggrecan and periostin) were examined on serial neighbouring sections throughout HH29 control (n = 9) and banded hearts (n = 7). *GAPDH* was used as an experimental control (all hearts expressed normal levels; Fig. 3Ae–f’).

**Fig. 2:**
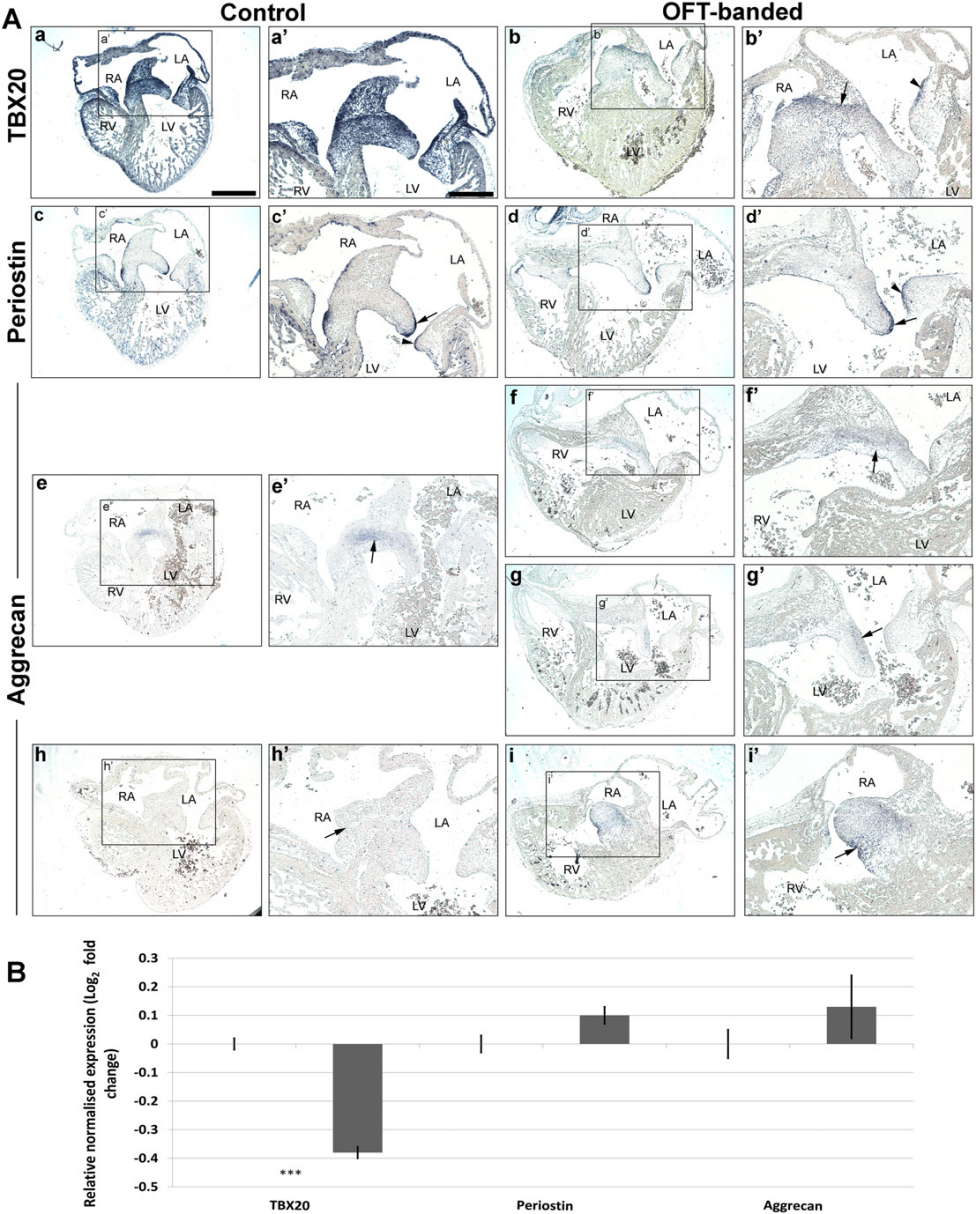
Expression of early extracellular matrix markers characteristic of valve primordia differentially expressed at HH29 upon alteration of haemodynamics. A. (a–b’) A marked reduction of expression of *TBX20* was observed in the entire endocardial cushion and the atrial and ventricular myocardium of the banded hearts (b, b’) compared to control (a, a’). However, distinct *TBX20* expression was seen in the boundary of the cushion (arrow in b’) and in the upper lining of the mitral valve (MV) (arrowhead in b’). (c–d’) No differential expression of periostin was found; expression was predominantly detected in the endocardial lining of the MV septal (arrow) and lateral (arrowhead) leaflets in both controls (c, c’) and banded hearts (d, d’). (e–i’) Aggrecan was expressed in the core of the endocardial cushion in control (e, arrow in e’) but not in the posterior part of the cushion (h, arrow in h’). Similar expression was seen in the central cushion in the banded hearts (f, arrow in f’). However, ectopic expression of aggrecan was also detected; aggrecan was seen in the atrialis side of the primordial MV (g, arrow in g’) and the lateral part of the tricuspid valve cushion on consecutive sections (i, i’). Arrow indicates the most intense expression of aggrecan at the borders of the cushion (i’). n = 9 for control and n = 7 for OFT-banded hearts. a’–i’ are the boxed areas in a–i respectively. Scale bar: 300 μm for Aa–I, 150 μm for Aa’–i’. LA, left atrium; LV, left ventricle; RA, right atrium; RV, right ventricle. B. Relative expression of early extracellular matrix genes characteristic of valve primordia was quantified by qPCR at HH29. *TBX20* was significantly downregulated in the banded hearts by 0.39 fold (***P < 0.001) compared to controls. Periostin and aggrecan mRNA levels were not significantly different between groups. Student's *t*-test (two-tailed). Data are mean ± s.e.m. (n = 5).

*TBX20* was expressed intensely in the EC, all four primordial AV valve leaflets and in part of the atrial and ventricular myocardium in the controls (Fig. 2Aa, a’), as previously reported [49]. In contrast, a marked reduction of *TBX20* expression was seen throughout the heart, including the cushion and all four primordial valve leaflets in banded hearts (n = 5/7; Fig. 2Ab, b’). However, *TBX20* expression was still distinct in the boundary of the EC subjacent to the atrial septum (arrow in Fig. 2Ab’) and also delineated in the upper lining of the lateral leaflet of primordial MV (arrowhead in Fig. 2Ab’). The qPCR quantified mRNA level of *TBX20* (n = 5 per group) demonstrated a 0.38-fold decrease (two-tailed Student's *t*-test; *P* < 0.001; Fig. 2B).

Consistent with the literature, punctuate periostin mRNA expression was detected throughout the endocardium of both atria and ventricles as well as the ventricular trabeculae in controls (data not shown) [31], [50]. Expression was further restricted to the endocardial lining of the atrialis side of the primordial MV septal (Fig. 2Ac, arrow in Fig. 2Ac’) and lateral (arrowhead in Fig. 2Ac’) leaflets. Additionally, abundant periostin mRNA was observed in the chordae tendinae of the left ventricular AV junction (data not shown). However, periostin mRNA was not found differentially expressed in the banded hearts by ISH or qPCR (Fig. 2Ad, d’, B).

At HH29, aggrecan was found localized in the cushion core, subjacent to the atrial septum in the controls (Fig. 2Ae, e’), but not in the posterior part of the EC (Fig. 2Ah, h’) or to the valve primordia (Fig. 2Ae, e’, h, h’). Similarly, aggrecan was also expressed in the central cushion in the banded hearts (Fig. 2Af, f’). However, ectopic aggrecan expression was found in five banded hearts (n = 7) in the lateral part of the TV cushion (Fig. 2Ai, i’), and in the atrialis side of the primordial MV leaflet (Fig. 2Ag, g’). qPCR revealed no significant differences in aggrecan expression (Fig. 2B).

### 3.3 Altered expression of shear stress responsive genes *KLF2* and *EDN1* in the OFT-banded heart at HH29

**Fig. 3:**
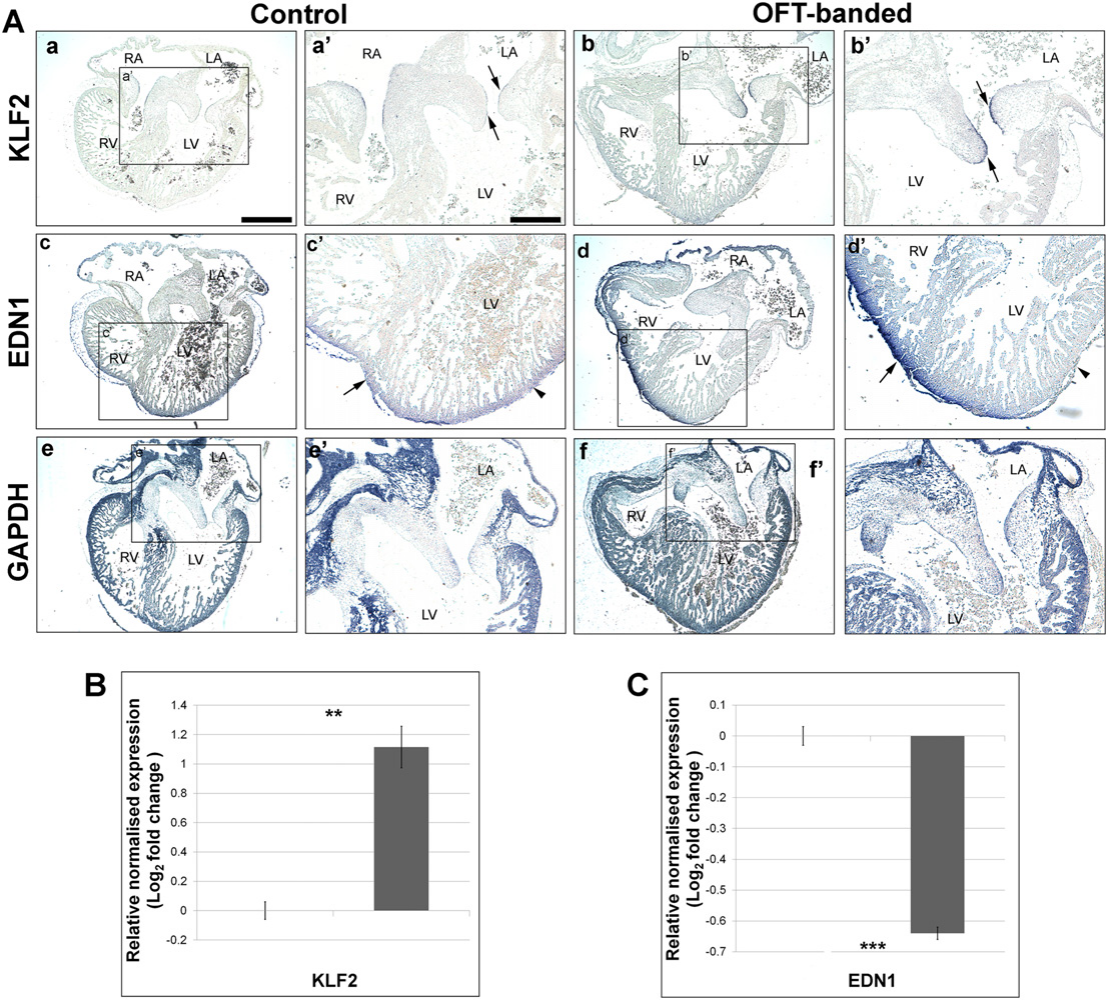
Altered shear stress gene expression in OFT-banded hearts. A. An increase of the shear stress responsive genes, *KLF2,* was detected in the primordial mitral valve endocardial lining of both septal and lateral leaflets (arrows) in banded (b, b’) compared to control hearts (a, a’). In one banded heart (n = 1/7), a noticeable decrease of *EDN1* was found in the compact myocardium of the outer curvature of the left ventricle (d, arrowhead in d’) but an increase was found in the right ventricle (d, arrow in d’). In contrast, *EDN1* was uniformly expressed in the compact myocardium of both ventricles in controls (c, arrow and arrowhead in c’). Experimental control *GAPDH* showed ubiquitous expression in both control and banded hearts (e, e’ and f, f’). n = 9 for control and n = 7 for OFT-banded hearts. a’–f’ are the boxed areas in a–f. Scale bar: 300 μm for Aa-f, 150 μm for Aa’–f’. LA, left atrium; LV, left ventricle; RA, right atrium; RV, right ventricle. B & C. Relative expression of shear stress responsive genes was determined by qPCR in HH29 control and OFT-banded hearts. A significant upregulation of *KLF2* was found (1.15 fold) in banded hearts (**P < 0.01) (B). Downregulation of *EDN1* by 0.65 fold was found in the OFT-banded hearts (***P < 0.001) (C). Two-tailed Student's *t*-test; data indicate mean ± s.e.m. (n = 5).

To investigate whether an abnormal primordial AV valve is associated with altered shear stress expression, the expression of known shear-stress responsive genes (*KLF2* and *EDN1*) [13] were analysed on neighbouring sections of banded and control hearts. *KLF2* in banded hearts showed a more intense and distinct expression at the endocardial lining of the atrialis side of the primordial MV septal and lateral leaflets (n = 3/7; arrows in Fig. 3Ab, b’) compared to controls (n = 7; Fig. 3Aa, a’). A significant 1.15-fold increase of *KLF2* was shown in the banded hearts (n = 4 per group; two-tailed Student's *t*-test; *P* < 0.01; Fig. 3B).

In control hearts, mRNA expression of *EDN1* was detected in the lining of the outer curvature of the right and left ventricular myocardium (n = 4; Fig. 3Ac, c’). However, the expression of *EDN1* was asymmetrical in one of the banded hearts (n = 1/7), with expression increased in the RV compact myocardium (arrow in Fig. 3Ad, d’) but was barely discernible in the LV ventricular myocardium (arrowhead in Fig. 3Ad’); the remaining banded hearts showed a general decrease of *EDN1* expression in both ventricles (n = 4/7). This was further confirmed by significant downregulation of 0.65 fold of *EDN1* mRNA (n = 5; *P* < 0.001; Fig. 3B). *GAPDH* was used as a positive control; ubiquitous expression patterns were seen in both control (Fig. 3Ae, e’) and OFT-banded hearts (n = 7 per group; Fig. 3Af, f’).

### 3.4 Reduced apoptosis in cushion and primordial AV valves in HH29 OFT-banded hearts

**Fig. 4:**
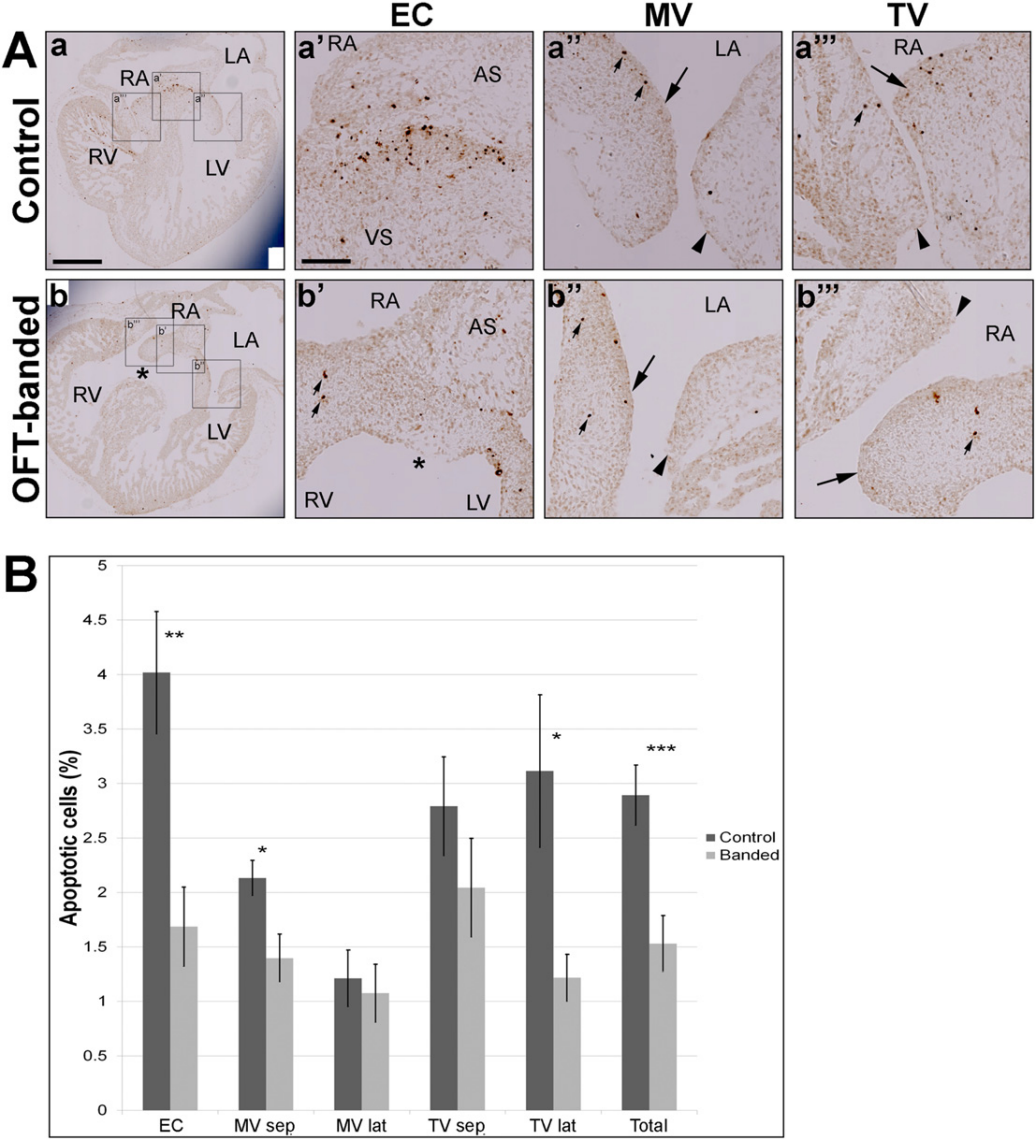
Apoptosis reduction in the primordial atrioventricular valve region of OFT-banded hearts. A. Apoptotic cells were detected by TUNEL in the endocardial cushion (EC), primordial MV leaflet and primordial TV valve leaflet (a & b). Clustering of apoptotic cells was identified in the EC area (area between atrial septum [AS] and ventricular septum [VS] in a’) in control, but few apoptotic cells in the EC area (between AS and asterix in b’; small arrows) in banded hearts. Otherwise, the apoptotic cells were found isolated and scattered (small arrows) in the primordial MV and TV (septal [denoted by arrow] and lateral leaflets [arrowheads] in both controls (a” and a”’) and banded (b” and b”’). Asterix in b and b’ denotes the ventricular septal defect in banded heart. Scale bar: 500 μm for Aa-b, 100 μm for Aa’–a”’ and Bb’–b”’. B. Apoptotic levels in sham and banded hearts at HH29. A decrease of apoptotic cells was shown in all areas analysed in primordial atrioventricular region (**P < 0.01). This was attributed to a significant decrease of apoptosis found in the EC (**P < 0.01), MV septal leaflet and TV lateral leaflet (*P < 0.05) in banded hearts compared to controls. No significant difference was found in the MV lateral leaflet and TV septal leaflet (*P* = 0.73 and *P* = 0.28 respectively). Student's *t*-test (two-tailed). Data indicate mean ± s.e.m. (n = 5). AS, atrial septum; EC, endocardial cushion; LA, left atrium; LV, left ventricle; MV, mitral valve; MV lat, mitral valve lateral leaflet; MV sep, mitral valve septal leaflet; RA, right atrium; RV, right ventricle; TV, tricuspid valve; TV lat, tricuspid valve lateral leaflet; TV sep, tricuspid valve septal leaflet; VS, ventricular septum.

To provide mechanistic insights into the dysmorphic valve primordia in banded hearts, an apoptotic study was performed. A cluster pattern of apoptotic cells was found in the EC region subjacent to the atrial septum in control (Fig. 4Aa’) but not OFT-banded hearts (Fig. 4Ab’). In contrast, apoptotic cells occurred singly and scattered in the primordial MV (Fig. 4Aa”, b”) and TV leaflets (Fig. 4Aa”’, b”’) in both groups.

The most evident apoptotic region was the EC; 4.02 ± 0.56% apoptotic cells were found in control hearts (Fig. 4Aa’), but only 1.69 ± 0.37% in the banded hearts (two-tailed Student's *t*-test; *P* < 0.01; Fig. 4Ab’, B). In primordial MV septal leaflets, 1.40 ± 0.22% of cells in banded hearts (arrow in Fig. 4Ab”) were undergoing apoptosis compared to 2.13 ± 0.16% in controls (Fig. 4Aa”, B; *P* < 0.05). Further, apoptotic cell numbers in the right lateral leaflet were significantly lower in the OFT-banded hearts (arrowhead in Fig. 4Ab”’), with 1.22 ± 0.22% apoptotic cells compared to 3.12 ± 0.7% in controls (Fig. 4Aa”’, B; *P* < 0.05). However, the primordial TV septal (arrow in Fig. 4Ab”’, B) and the primordial MV lateral leaflets (arrowhead in Fig. 4Ab”, B) showed a non-significant reduction of apoptotic cells in the banded hearts compared to controls (arrow in Fig. 4Aa”’, B and arrowhead in Fig. 4Aa”, B, respectively; *P* > 0.05). The total apoptotic cells identified in the primordial valve region was significantly lower in banded hearts (1.53 ± 0.26%) compared to controls (2.89 ± 0.28%; *P* < 0.01; Fig. 4B).

### 3.5 Spatial-temporal expression profiles of ECM valve proteins

**Fig. 5:**
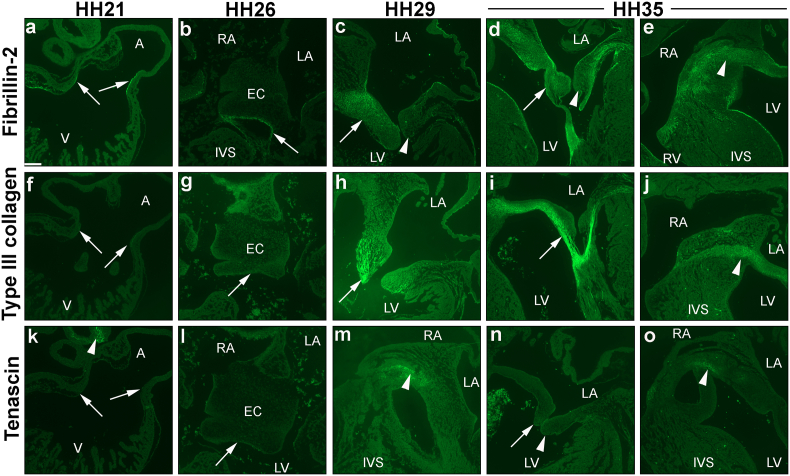
Cardiac spatial-temporal expression profile of fibrillin-2, type III collagen and tenascin. Chick hearts were isolated at HH21 (a, f, k), HH26 (b, g, l), HH29 (c, h, m) and HH35 (d, e, i, j, n, o), with staining for fibrillin-2 (a–e), type III collagen (f–j) and tenascin (k–o). Fibrillin-2 was expressed in the endocardium lining at the atrioventricular canal (AVC) at HH21 (a), whereas staining for type III collagen and tenascin was not observed at the AVC (arrows in f & k respectively) or in any parts of the developing heart besides the pharyngeal arches (arrowhead in k). At HH26, only fibrillin-2 was seen at the endocardial lining of the cushion mesenchyme (b); type III collagen and tenasin were negative (arrow in g & l). At HH29, fibrillin-2 and type III collagen were observed in the primordial mitral valve (MV) septal leaflet (arrow in c & h respectively; arrowhead in c denotes negative lateral leaflet). Tenascin expression was localized to the endocardial cushion (EC) at HH29 (arrowhead in m). At HH35, fibrillin-2 (d) and type III collagen (i) demonstrated positive staining in the primordial MV leaflet, with the most intense immunostaining seen in the chordae tendinae (arrow). Staining of tenascin remained negative in the primordial MV septal and lateral leaflets (arrow and arrowhead in n respectively). Widespread expression of fibrillin-2 (arrowhead in e) and type III collagen (arrowhead in j) was also observed in the central cushion. Tenascin expression was only seen in the cushion (o). A, atria; IVS, interventricular septum; LA, left atrium; LV, left ventricle; RA, right atrium; RV, right ventricle; V, ventricle; n = 2 per stage. Scale bar: 120 μm.

With regards the expression of ECM proteins during early AV valvulogenesis, fibrillin-2 has partially been studied within the literature in the chick [48], whereas the expression of both type III collagen and tenascin have only been studied in other species [51], [52]. Therefore, immunohistochemical analyses were performed to fill these gaps in knowledge with a spatial-temporal expression analyses at crucial stages of early chick valvulogenesis (HH21, HH26, HH29 and HH35; n = 2 per stage). Fibrillin-2 was detected at all stages. At HH21, distinct fibrillin-2 immunoreactivity was seen at the endocardial lining of the AVC (Fig. 5a), and at HH26 was restricted to the border of the fused mesenchymalized EC (Fig. 5b). Positive immunostaining was not seen in any parts of the HH21 and HH26 developing heart with type III collagen (Fig. 5f, g) and tenascin (Fig. 5k, l). At HH29, stronger staining of fibrillin-2 was visible in the primordial MV leaflet (arrow in Fig. 5c), with little to no immunoreactivity of fibrillin-2 in the left lateral leaflet (arrowhead in Fig. 5c). At HH35, immunolocalisation of fibrillin-2 was seen in a restricted pattern in both septal and lateral leaflets, and in the chordae tendinae (Fig. 5d). A fairly strong immunostaining of fibrillin-2 was seen in the central endocardial cushion at this stage (arrowhead in Fig. 5e). Type III collagen was seen to a lesser degree in the HH29 primordial MV leaflet, being more centralized at the tip of the valve leaflet (Fig. 5h). However, strong staining of type III collagen was found extending from the septal leaflet to the chordae tendinae at HH35 (Fig. 5i), and in the core cushion (Fig. 5j). Positive staining of tenascin was detected in the core cushion at HH29 (Fig. 5m) and HH35 (Fig. 5o), but not in the primordial MV leaflets (Fig. 5n).

### 3.6 Decreased expression of ECM proteins in primordial AV valve in HH35 OFT-banded hearts

During post-EMT stages, the cushion mesenchyme normally differentiates into valve interstitial cells, the specialized valvular fibroblasts that express fibrillary collagens, and chondroitin sulfate proteoglycans [3], [34]. Therefore, in order to determine whether valve primordia were affected at later stage of valve development, type III collagen and tenascin immunohistochemistry were performed, as well as differentiation marker fibrillin-2 [48].

**Fig. 6:**
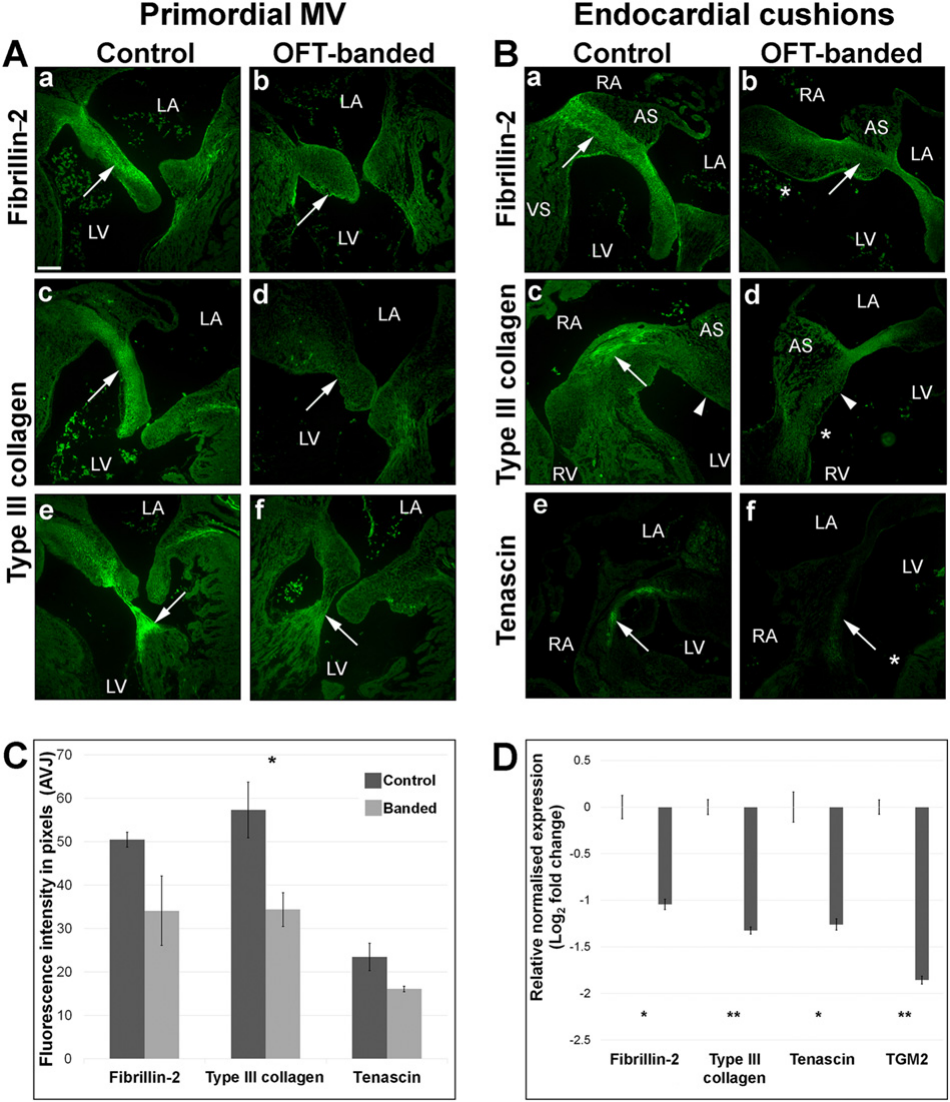
Decreased expression of extracellular matrix markers characteristic of valvulogenesis in HH35 OFT-banded hearts. A. Decreased and a more diffuse staining of fibrillin-2 (arrow in b) in the primordial MV septal leaflet compared to controls (a). A similar reduction of type III collagen expression was also seen in the septal leaflet (arrow in d) and the chordae tendinae (arrow in f) in OFT-banded compared to controls (c and e, respectively) (n = 5). B. Positive expression of fibrillin-2 (a), type III collagen (c) and tenascin (e) in the endocardial cushion (denoted by an arrow) seen in the controls was decreased in banded hearts (arrow in b, d and f respectively; asterix denotes ventricular septal defect). Arrowhead in c and d denote equivalent region of the endocardial cushion between controls and banded hearts (n = 5). C. The fluorescence signal intensity of the primordial mitral valve leaflet was semi-quantified using MetaExpress software, with a significant reduction of fluorescence intensity for type III collagen (two-tailed Student's *t*-test; *P* < 0.05; n = 5). Scale bar in Aa 120 μm; same magnification for all panels in A&B. D. qPCR showed a 1.06- and 1.26 downregulation of fibrillin 2 and tenascin C in HH35 banded hearts. A significant decrease was seen in type III collagen and *TGM2* by 1.32 and 1.85 fold, respectively (two-tailed Student's *t*-test; *P* < 0.01; n = 3). Data represent means ± sem. AS, atrial septum; AVJ; average grey level. LA, left atrium; LV, left ventricle; RA, right atrium; RV, right ventricle; VS, ventricular septum.

Fibrillin-2 and type III collagen immunostaining were detected strongly throughout the leaflet in controls (n = 5; Fig. 6Aa, c respectively). A noticeable weaker and more diffuse staining of fibrillin-2 (n = 3/5) and type III collagen (n = 4/5) was seen in the truncated primordial MV leaflets in the OFT-banded hearts (Fig. 6Ab, d). Likewise, a reduction of type III collagen (n = 4/5) was seen in the chordae tendinae in the banded hearts (Fig. 6Af) when compared to controls (Fig. 6Ae). Similarly, a positive staining was seen in the central endocardial cushion area subjacent to the atrial septum with zonal restriction of fibrillin-2 in controls (Fig. 6Ba), with decreased immunolocalisation in the core cushion of banded hearts (n = 3/5; arrow in Fig. 6Bb). The positive staining of type III collagen was located in the central cushion in controls (arrow in Fig. 6Bc). In contrast, little to no immunoreactivity of type III collagen was observed in the OFT-banded hearts (n = 4/5; arrowhead denotes equivalent region of the cushion in Fig. 6Bc, d). Lastly, tenascin staining was in the centre of the cushion of controls (Fig. 6Be) but reduced in all banded hearts (n = 5; arrow in Fig. 6Bf). The fluorescent intensity was further quantified in the primordial MV leaflets in fibrillin-2, type III collagen and tenascin (Fig. 6C; n = 5). The average grey level of fibrillin-2 was 50.48 ± 1.71 in controls but 34.11 ± 8.02 in banded hearts, a 32.4% decrease of fluorescent signal. However, this reduction was not significant (*P* > 0.05). A non-significant 30.4% decrease of fluorescence intensity was seen for tenascin, where banded hearts and control presented 16.1 ± 0.63 and 23.48 ± 3.14 respectively. The fluorescence intensity of type III collagen was 57.31 ± 6.39 in controls and 34.37 ± 3.92 in banded hearts, a significant reduction of 40% (two-tailed Student's *t*-test; *P* < 0.05). In order to support the decreased expression of these ECM proteins, mRNA level was quantitated by qPCR at HH35 isolated valve region. *FBN2* and *TNC* both showed a significant downregulation of 1.06 and 1.26 fold (n = 4 per group; two-tailed Student's *t*-test; *P* < 0.05; Fig. 6D). Further, *COL3A1* and *TGM2* demonstrated a significant downregulation of 1.32 and 1.85 fold in banded hearts (n = 4 per group; *P* < 0.01; Fig. 6D).

### 3.7 Morphologically normal endocardial cushions and atrial septation in HH26 OFT-banded hearts

Atrial septation was seen to be morphologically normal in banded hearts (n = 5), a process which completes around HH24 [28]. However, the studies described above suggested that the defective valve primordia at HH29 and HH35 might form from a deformed EC. In order to examine this, ISH was performed on markers of the EC area on neighbouring sections at HH26 (post-EMT).

**Fig. 7:**
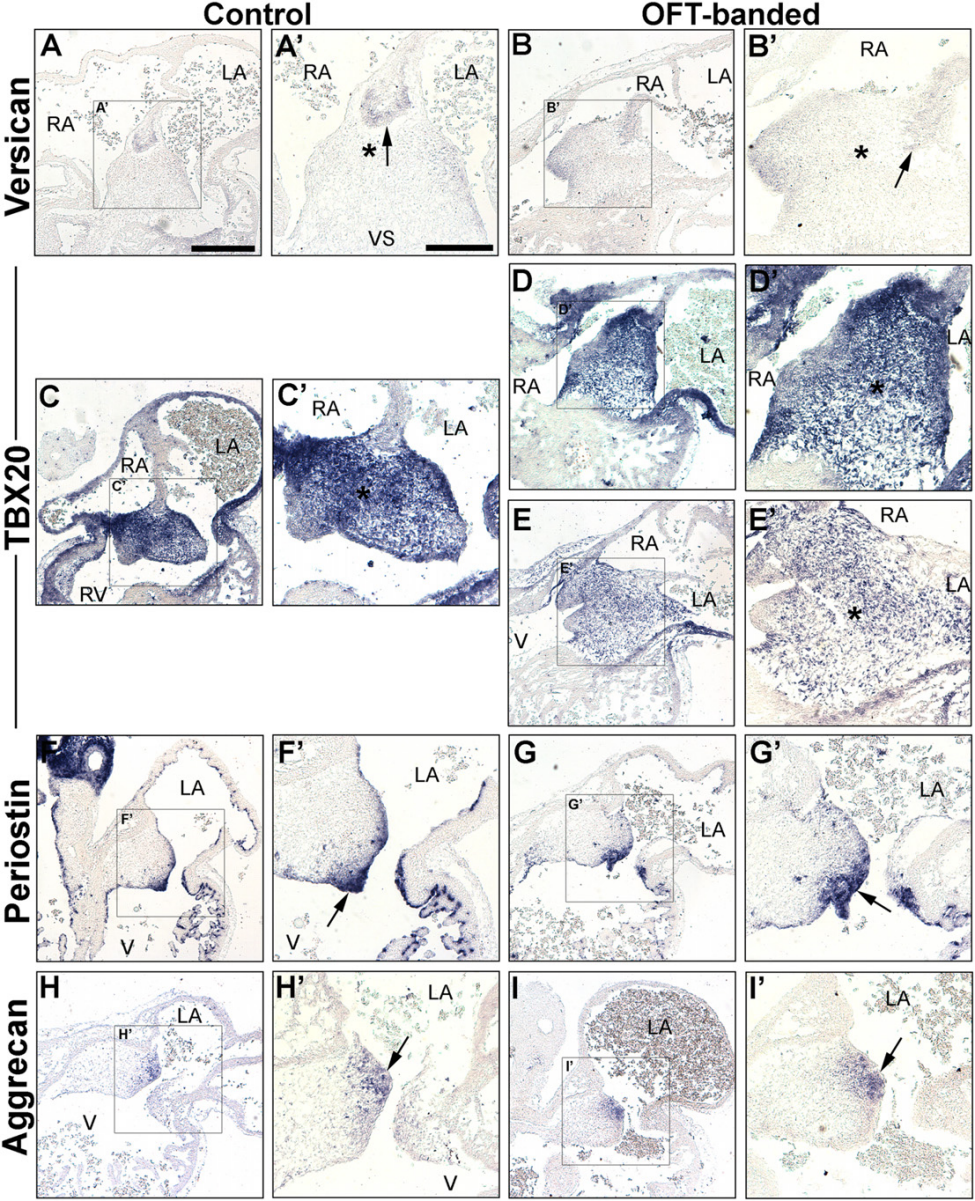
Normal atrial septation with mild differential expression of an endocardial cushion marker in haemodynamically altered hearts at HH26. A–B’: Normal atrial septation (mesenchymal cap [arrow] fused with the AV endocardial cushions [asterix]) was seen, along with expected immunoreactivity for versican, in control (A, A’) and banded (B, B’) hearts. C–E’: Strong *TBX20* expression was observed in the entire endocardial cushion area in controls (C, C’) and in three banded hearts (D, D’). In contrast, decreased expression in the endocardial cushions in two banded hearts (E, E’) was seen compared to controls (C, C’). The endocardial cushion area is denoted by an asterisk (C’, D’, E’). F–G’: Periostin was intensely expressed in the endocardial lining of the cushion in both control (F, F’) and banded hearts (G, G’) (arrow in F’ and G’). H–I’: Expression of aggrecan was seen in the subatrial region of the endocardial cushion in both control (H, arrow in H’) and OFT-banded hearts (I, arrow in I’) n = 5 per group. A’–I’ are the boxed areas in A–I respectively. LA, left atrium; RA, right atrium; RV, right ventricle; V, ventricle.

As expected, versican was expressed in the mesenchymal cap of the atrial septum that fused with the AV cushions in both controls (arrow in Fig. 7A, A’) and OFT-banded hearts (n = 5 per group; Fig. 7B, B’), confirming normal atrial septation had occurred [53]. *TBX20* was strongly expressed in the EC and part of the ventricular myocardium (Fig. 7C, C’) of controls. Less intense *TBX20* expression was detected on serial sections in two of the banded hearts analysed (2/5; Fig. 7E, E’) compared to controls. Otherwise, *TBX20* expression was found normal (3/5; Fig. 7D, D’). At HH26, abundant periostin mRNA was detected in the endothelial lining and adjacent tissue of the AV cushions in controls (Fig. 7F, F’) and OFT-banded hearts (n = 5; Fig. 7G, G’). Aggrecan expression was restricted to the lateral part of the left side of the endocardial cushion in both groups (arrow in Fig. 7H, H’, I, I’).

## 4 Discussion

Valve defects constitute an important medical issue challenging our society. Though valve developmental defects may initially be asymptomatic, they are often progressive and contribute to valve disease later in life. Abnormal valves often display dysregulation of ECM proteins such as collagens and glycosaminoglycans, as seen in patients with myxomatous mitral valve and mitral valve prolapse [54]. Structural changes of ECM are associated with the aberrant re-expression of early valve mesenchymal markers, implying the reactivation of the fetal gene program that is normally quiescent in adulthood [55], [56].

Recently, valvulogenesis has been shown to be haemodynamically dependent, with occlusion of blood at either the inflow or outflow resulting in absent AV valve in zebrafish [10]. We delineated the role of abnormal haemodynamics in the developing primordial mitral AV valve and its alignment with other septal components at critical stages of chamber septation and valve development. A ligature was placed around the HH21 outflow region and harvested at HH26 (atrial septation complete, post-EMT with fusion of superior and inferior cushions), HH29 (fused cushions mature, primordial AV valve elongates and ventricular septation complete) and HH35 (remodelling). In this OFT-banding model, alteration of haemodynamics has previously been shown to result in increased ventricular pressure and wall shear stress, as well as higher peak blood flow velocity in the OFT region [57], [58], [59], [60]. Morphologically, the OFT-banded hearts exhibited right-shifted position of the OFT and appeared to be approximately 1.77 times larger than controls by HH29 (unpublished data; Fig. 1Cc, d), An enlarged heart is a characteristic of pressure-overloaded embryonic hearts [21], [58], [61]. However, the crown rump length and eye diameter (as described here) together with cardiac output and heart rate [57], [62], remained unchanged in this model.

The AV endocardial cushion was structurally normal at HH26 but dysmorphic and truncated primordial mitral valves were seen at HH29 and HH35, with a smaller primordial valve region. AV valves were generally immature in another altered haemodynamic model (vitelline vein clip) [20], with the tricuspid valve morphologically abnormal upon banding [21].

*TBX20* is important in promoting EC cell proliferation and migration, and ECM gene expression in vitro [43], [44]. In addition, *Tbx20* plays a role in EC maturation and valve elongation [41]. In normal development, fusion of the superior and inferior AV cushions and their subsequent union with the dorsal mesenchymal cap of the primary atrial septum has occurred by HH26 [26], [63]. *TBX20* expression was analysed in order to elucidate if it was differentially expressed in the morphologically normal OFT-banded HH26 early valve primordia. Interestingly, despite no cushion structural abnormalities in HH26 banded hearts, decreased expression of *TBX20* was observed in the EC region in two of the five banded hearts (asterisk in Fig. 7E, E’). Periostin and aggrecan were not differentially expressed in neighbouring sections. Periostin and aggrecan have been found downregulated in the *Tbx20* knockdown [41]. The decrease in expression of *TBX20* (− 0.39 fold) in our study at HH29 suggests alteration of haemodynamics leads to reduced *TBX20* and concurrent ectopic expression of its target aggrecan, which together engendered AV valve elongation defects. The data described here supports the hypothesis that *TBX20* acts upstream of aggrecan. Aggrecan is one of the main chondroitin sulfate proteoglycan constituents of the spongiosa layer in avian valves, and is required to withstand compressive forces against blood flow [32], [43], and reportedly has a role in valve cell differentiation [44], [64].

Periostin has been previously reported for its multifaceted role in cell migration and differentiation; it promotes differentiation of prevalvular mesenchymal cells into collagen-producing fibroblastic cells termed ‘valve interstitial cells’ while repressing transformation into myocyte lineages [31], [44]. Periostin is also able to interact with other ECM components such as collagen, tenascin and fibronectin [65], [66], [67]. Interestingly, although periostin was normally expressed, collagen III and tenascin were found to be differentially expressed. It can be speculated that mRNA downregulation of one transcript variant might lead to a compensatory upregulation of another variant upon altered haemodynamics, as differential periostin isoform expression profiles were observed in periostin-null mice after myocardial infarction [68], [69].

Remodelling of the AVC and OFT tissues occur through apoptosis [70]. Clustering of apoptotic cells is involved in cushion differentiation and outgrowth, as evidenced by a lack of apoptosis and overabundant cushions in the Nf-1 null mouse [71], [72]. In contrast, our study showed a decrease in apoptosis in the primordial AV region and smaller cushions in banded hearts. This suggests normal levels of apoptosis are required for EC differentiation [72]. In addition, the regulatory factors *Bmp4*, *Bmp2* and *Msx2* have been associated with cells undergoing apoptosis, and hence differentiation [73]. As *Bmp2* is considered to be a key downstream targets of *Tbx20* in AVC development [74], it is a gene worthy of future investigation.

At a later stage of valvulogenesis, cushion mesenchyme is normally differentiated into valve interstitial cells, cells which express genes that encode fibrillary collagens, chondroitin sulfate proteoglycans and elastin, which are associated with stratified ECM of mature valves [3], [34], [75]. Upon abnormal blood flow, the differentiation process was predicted to be affected in HH35 banded hearts, with weaker expression of *COL3A*, *TNC* and *FBN2* mRNA. Fibrillin-2 is known to be expressed in a subset of endothelial cells competent to transdifferentiate into cushion mesenchyme and connective tissue fibroblasts of the valve leaflets [48]. Also, *FBN2* transcripts accumulate prior to tissue differentiation, decreasing rapidly thereafter during development [76]. We speculate that the decrease of fibrillin-2 staining and its mRNA in our study suggests differentiation failed to occur. Similarly, in patients with mitral valve prolapse syndrome, a more diffuse and weaker staining pattern of fibrillin and type III collagen were seen in the area of myxoid degeneration of diseased MV leaflets [77]. Further, part of the EC is differentiated into valve leaflets and chordae tendinae during normal development, and the attachment of the papillary muscle to valve leaflets without chordae tendinae was seen in human fetal hearts at weeks 5–19 week, when differentiation failed to occur [78], [79]. Additionally, the downregulation in banded hearts of the gene transglutaminase-2 (*TGM2*), which encodes the TG2 ECM cross-linking protein, could lead to the decrease of the ECM proteins fibrillin-2 and type III collagen. TG2 has been reported for its role in ECM condensation and organization by interacting with filamin-A and serotonin [5]. TG2 has also been implicated in osteoblast differentiation, with its expression defining the border of differentiation; TG2 protein expression was absent in the cushion but present in the adjacent myocardium prior to cushion fusion [22], [80]. It was also in almost all the interstitial cells following cushion fusion (HH27) and maturation (HH35) [22]. Downregulation of *TGM2* in our study suggests aberrant differentiation of the cushion mesenchyme, indicating that the proteolytically activate protein could be an area of future interest.

Normal fluid flow in the heart is important in shaping the EC into the developing cardiac valve leaflets [10], [11]. Knockdown of *trpv4* (encodes a mechanotransduction protein), which is upstream of the shear stress responsive gene *klf2a*, led to the development of dysmorphic valves in zebrafish [81]. In addition, the *klf2a* null mutant zebrafish displayed an array of valvular phenotypes, attributed to cell disorganization at the AVC and impaired fibronectin synthesis [12]. In addition, laminar shear stress is involved in endothelial differentiation [82], [83]. In the OFT-banded heart, higher peak and end diastolic interventricular pressure was seen [18], [57], [58]. Further, increased peak blood flow velocities near the banding site and regurgitation around the AV region were found at HH27 [17], [84]. Alteration of wall shear stresses, as a result of changes of blood flow pattern, are known to alter shear stress responsive genes *KLF2* and *EDN1* [85], as did altering haemodynamics in the venous clip model [86]. Here, we support the notion of pressure overloading upon OFT-banding by showing the up- and down-regulation of the *KLF2* and *EDN1* shear stress genes respectively, in banded hearts. This might lead to aberrant differentiation of the EC cushion and decreased expression of the ECM proteins in the valve leaflet and hence a maturation arrest in valve development.

## 5 Concluding remarks

Fetal valvulogenesis consists of two main phases: EMT and post-EMT maturation, with the latter phase involving the remodelling of the cushion into mature valve leaflets. Defects arising in the post-EMT valve were associated with aberrant expression of early mesenchymal markers and dysregulation of ECM proteins such as fibrillin and collagen which were previously seen in patients with mitral valve prolapse syndrome, with the underlying mechanism remaining poorly understood. In this study we show aberrant expression of *TBX20*, aggrecan and shear stress responsive genes upon abnormal haemodynamics. The decrease in apoptosis and the dysregulation of ECM proteins indicate the mechanism. Together, our data suggest that failure of differentiation of cushion mesenchyme into valvular interstitial cells caused by abnormal blood flow, is a potential mechanism to give rise to valve defects post-EMT. Therefore, this study provides new insights into potential aetiologies of human congenital valve malformations.

## Funding

This work was supported by British Heart Foundation [grant number FS/12/44/29619 to S.L.].

## Disclosures

No competing interests declared.
